# From Identification to Resistance: Contemporary Insights into Candida Species Causing Vaginitis

**DOI:** 10.7759/cureus.108327

**Published:** 2026-05-05

**Authors:** Suman A Pawar, Geeta S Karande, Satish R Patil

**Affiliations:** 1 Department of Microbiology, Krishna Institute of Medical Sciences, Krishna Vishwa Vidyapeeth (Deemed To Be University), Karad, IND

**Keywords:** antifungal resistance, antifungal susceptibility testing, candida albicans, chromagar candida, non-albicans candida, vulvovaginal candidiasis

## Abstract

Vulvovaginal candidiasis (VVC) is among the most frequent reasons why vaginitis occurs in women and represents a significant clinical and public health concern worldwide. Although *Candida albicans* remains the predominant etiological agent, infections due to non-albicans speciesof* Candida *(NAC)like*C. glabrata, C. tropicalis, C. krusei,* and *C. parapsilosis* are increasingly reported, particularly in recurrent and complicated cases. The pathogenesis of VVC is multifactorial and influenced by host immune status, hormonal factors, disruption of the vaginal microbiota, and metabolic conditions such as diabetes mellitus, antibiotic use, and pregnancy. Emerging antifungal resistance, especially to azole drugs, poses a major challenge in the oversight of VVC and underscores the importance of accurate identification of species and susceptibility to antifungal testing. Chromogenic media and standardized in vitro susceptibility methods have improved rapid diagnosis and targeted therapy. This review highlights the epidemiology, risk factors, pathogenic mechanisms, virulence attributes, diagnostic approaches, and therapeutic challenges associated with VVC, with an emphasis on the growing clinical relevance of NAC species and antifungal resistance. Continued surveillance, improved diagnostics, and development of novel antifungal strategies are essential to optimize patient outcomes and reduce disease burden.

## Introduction and background

Numerous bacterial, protozoal, fungal, and ectoparasitic parasites can cause reproductive tract infections, which can be communicable or non-communicable [1]. Renal tract infections (RTIs) are a serious health risk since they result in widespread death and morbidity in women, especially during reproductive age, especially in underdeveloped nations where RTIs are common [1]. *Candida albicans* is the primary cause of vulvovaginal candidiasis (VVC); however, episodes caused by non-albicans species of *Candida *(NACs)* *seem to be on the rise in both healthy and immunodeficient women [2]. The non-albicans species that are most commonly implicated are* C. glabrata, C. tropicalis, C. krusei, *and *C. parapsilosis* [2]. Certain risk groups, such as women with diabetes, pregnant women, and those on antibiotics or oral contraceptives, have a greater chance of acquiring *Candida *vaginitis [3]. VVC is commonly described as 'white cottage cheese' discharge per vagina, accompanied by irritation of the vulva and vagina [4]. Protocols for quick identification and in vitro screening for antifungal sensitivity have been established to respond to the growth in frequency of VVC caused by NACs, with a rise in resistance to commonly used antifungals [5]. This narrative review aims to summarize current evidence on *Candida *species and antifungal resistance in VVC.

## Review

Methodology

*Search Strategy*****

This study is a PRISMA-based narrative review. A thorough search of the literature was carried out to identify studies addressing species identification of *Candida *isolates in vaginitis and their antifungal susceptibility testing. Search engines such as Google Scholar, PubMed, and ScienceDirect were searched. The search strategy employed a mix of MeSH terms and free-text keywords using Boolean operators (AND, OR). Search terms included 'vaginitis', 'vulvovaginal candidiasis', '*Candida* species', '*Candida albicans*', 'non-albicans *Candida*', 'species identification', 'antifungal susceptibility testing' (AFST), and 'antifungal resistance'.

Selection and Screening Process

**Figure 1:**
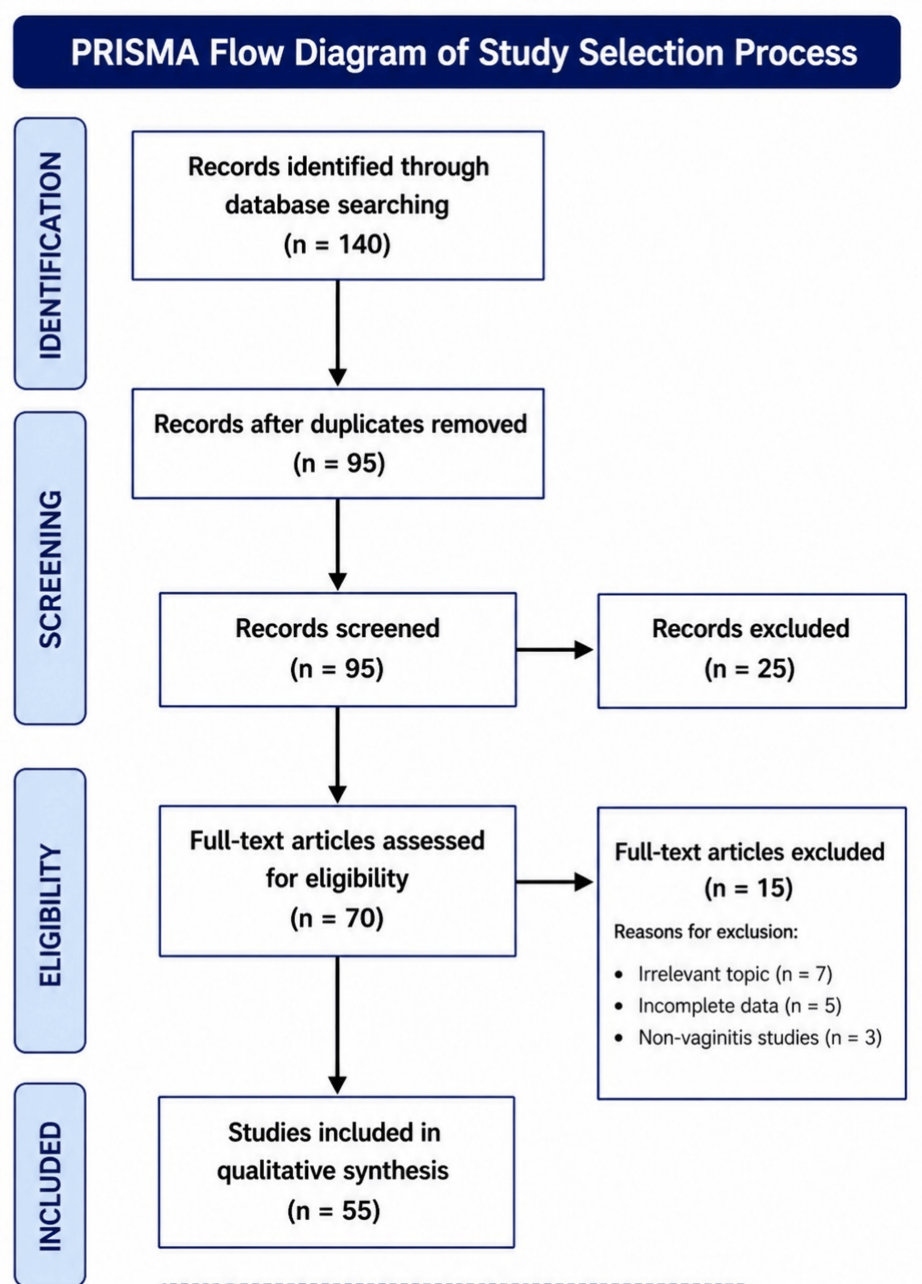
Article selection and screening process. Source: Created by the author based on a literature search strategy. n: number of articles.

Articles were screened based on titles and abstracts, followed by a full-text review of relevant studies. The initial database review produced 140 results for analysis. A total of 95 articles remained after duplicate papers and irrelevant content were removed from the initial 140 papers identified. The final review incorporated 55 articles that passed through identification, selection, and inclusion and exclusion criteria evaluation processes, starting from 70 initially assessed articles. Findings were synthesized to describe trends in *Candida* species distribution, identification techniques, and antifungal susceptibility patterns. A schematic representation of the literature selection process is shown in Figure 1.

Inclusion Criteria

Only English language articles published were included. Original research articles and review articles reporting VVC and *Candida *species identification from vaginal specimens or AFST were eligible.

Exclusion Criteria

Case reports with insufficient data, animal studies, studies not related to vaginitis or *Candida *infection, and Duplicate articles were excluded.

Background

The complex network of organs that make up the female reproductive system can be split into internal and external genitalia [6]. The labia majora and minora, vestibule, Bartholin glands, Skene glands, clitoris, mons pubis, perineum, urethral meatus, and periurethral region are among the components outside of the actual pelvis that make up the external genitalia [6]. The structures found inside the actual pelvis, such as the uterus, fallopian tubes, ovaries, vagina, and cervix, are known as internal genitalia [6]. Sebaceous glands, sweat glands, Bartholin glands, and skene glands are the primary sources of normal vaginal secretions [7]. Based on the type of epithelium and other elements in the microenvironment, the vagina, ectocervix, and endocervix are all vulnerable to different diseases. *Candida *species can infect the stratified squamous epithelium of the vagina and ectocervix [8]. The main steroid hormone controlling the female reproductive system is oestrogen [9]. In the vagina, presence or absence of oestrogen, such as during menopause, affects the growth of epithelial mucosa cells [9].

The vagina is a fibromuscular canal that is between 6 and 8 cm long. Anatomically, it is situated anteriorly to the rectum and posteriorly to the urethra and bladder wall. The front wall is roughly 6 cm shorter than the posterior wall, which is around 8 cm, due to the vagina's oblique shape. The cervix forms a canal between the two structures as it expands into the vagina. The cervix is thus separated into a supravaginal and a vaginal portion [6] Among the most prominent gynaecological conditions is vaginitis, which is an inflammation of the vaginal mucosa and submucous connective tissues [10]. An imbalance of good bacteria in the vagina may cause vaginitis, which manifests as changes in the quantity, colour, or smell, irritation, or itching or burning [11]. In the clinic, bacterial vaginosis (BV), VVC, aerobic vaginitis (AV), and trichomonas vaginitis (TV) are the most prevalent forms of vaginitis [10].

VVC is an infection that mostly affects the oestrogenized vagina and vestibule [12]. The proliferation of species of *Candida *in the vagina is the cause of VVC, also known as moniliasis [13]. Based on clinical presentation, host characteristics, microbiology, and treatment response, VVC is categorized as either an uncomplicated or severe condition [14]. A significant percentage of women with VVC has simple vaginitis. This manifests as infrequent instances of mild to severe infections, mostly caused by *C. albicans*, which primarily affect healthy adult women without any risk factors [14]. Around 10-20% of women have complicated VVC, which is characterized by more severe attacks or is caused by NAC species with implications for diagnosis and treatment [14]. Three or more episodes of vaginitis per year are referred to be recurrent vulvovaginitis (RVV) [15]. Recurrent vaginitis is frequently caused by infectious organisms as well as unsanitary habits, poor menstrual care, having several sexual partners, and contraceptive trends [15].

Epidemiology of VVC

Overall, in Delhi, the incidence of vaginal secretions is 29.9%, while in India overall, it is 30% [15]. Every year, around 5-10 million women worldwide seek gynaecologic care for vaginitis [16]. VVC affects 70-75% of women at least once throughout their lifetime; 40-50% of these cases will recur, and 5-10% will have recurrent VVC, which is defined as four or more episodes annually [17]. It is believed that 20% of healthy women have asymptomatic *C. albicans *carriage and as high as 30% in expectant mothers [18,19]. VVC affects over two-thirds of women at least once over their lifetime, and nearly half of them have multiple episodes [13]. Because VVC is linked to both direct and indirect economic expenses, HIV and other sexually transmitted illnesses, and ascending genital tract infections, it has been acknowledged as a global issue of concern [16]. Isolates of *C. albicans* have been found in food, soil, animals, and medical settings. Non-albicans species can also be found in animal habitats. Just 15 of the 150 species of *Candida *that are known to exist have infected people. *C. albicans, C. glabrata, C. tropicalis, C. parapsilosis, C. krusei, C. guilliermondii, C. lusitaniae, C. dubliniensis, C. pelliculosa, C. kefyr, C. lipolytica, C. famata, C. inconspicua, C. rugosa, *and *C.* *norvegensis *are among these pathogens [20].

Infections brought on by NACs have increased, despite* C. albicans* becoming the highest often diagnosed infectious species of *Candida *globally [21]. In low- and middle-income nations like India, the exorbitant cost of echinocandins prevents their widespread use. Because Amphotericin B (AMB) is nephrotoxic, it is only used in severe cases of invasive candidiasis. As a result, fluconazole is now used more frequently to treat candidiasis. Multidrug-resistant species, such as *C. glabrata, C. krusei, *and *C. auris*, have emerged as an outcome of this reliance on a small range of antifungal medications [21]. Since its first discovery in Japan, *C. auris* infection has been documented in 47 nations, including India. The kind of* Candida* infection might also be influenced by age. Compared to adults, *C. parapsilosis* is more frequently isolated from newborns and babies. In India, a large number of clinical reports about the diagnosis, treatment, and results of candidiasis have been published. The aetiology and clinical manifestation of candidiasis depend on the patient's age, organs affected, medical history, and geographic location. Therefore, it's critical to evaluate and understand how *Candida *infections are changing in India [21].

While a lot of research has been done to discover the pathogenic characteristics in *C. albicans*, comparatively little is known about NAC species [22]. Additionally, the aetiological profile differs between nations as well as regions within a single nation [23]. Geographical location, patient cohort, and prior exposure to antifungal medications can all affect the relative distribution of each species of *Candida *[24]. In contrast to the majority of fungal infections, *C. albicans* is typically thought to be obligately linked to warm-blooded mammals [25].

Risk factors

As shown in Table 1, numerous factors, including host factors, local defence systems, gene polymorphisms, allergens, serum glucose levels, antibiotics, psychological stress, oestrogens, and sexual activity, can either initiate or enhance the condition [12].

**Table 1:** Potential risk factors of vulvovaginal candidiasis (VVC). Source: Created by the author based on the reviewed literature. HRT: hormonal replacement therapy; PID: pelvic inflammatory disease.

Risk factor	Mechanism/Clinical association	
Host and local defence factors [12]	Alterations in host immunity and vaginal defence mechanisms predispose to *Candida *overgrowth	
Genetic polymorphisms [12]	Influence host susceptibility to *Candida *colonization	
Allergens [12]	Trigger inflammatory responses, enhancing disease severity	
Elevated serum glucose levels [26]	Promotes *Candida *adherence and growth in vaginal epithelial cells	
Diabetes mellitus [26]	Increased incidence of vaginal *Candida *infections due to hyperglycaemia	
Oestrogen levels [12]	Reduced oestrogenization lowers *Candida *colonization in premenarchal and postmenopausal women not on hormonal replacement therapy (HRT)	
Sexual activity [12]	May facilitate mechanical transfer and mucosal disruption	
Psychological stress [12]	Contributes to immune dysregulation and *Candida *overgrowth	
Untreated vaginal candidiasis [27]	May progress to pelvic inflammatory disease (PID), infertility, congenital neonatal infection, chorioamnionitis, abortion, and prematurity	
Menopause (senile vaginitis) [28]	Reduced oestrogen, glycogen, and Lactobacilli favour pathogenic bacterial and fungal invasion	
Antibiotic usage [29]	Disrupts vaginal microbiota, reduces *Lactobacillus *species, and promotes *Candida *overgrowth	
Antibiotic overuse [29]	Leads to development of resistant strains	

Pregnancy represents a high-risk state for vaginal candidiasis, as summarized in Table 2. The issue is more severe during pregnancy since *Candida *colonization is linked to premature birth and infant death, and up to 65% of pregnant women can contaminate their unborn children, which can lead to invasive neonatal candidiasis [30]. A woman's immune system is suppressed as a result of increased emotional stress during childbearing. In the end, the overgrowth of *Candida *species is accelerated by the compromised immune system and turns into a pathogen [31].

**Table 2:** Potential risk factors of VVC throughout pregnancy. Source: Created by the author based on the reviewed literature. VVC: vulvovaginal candidiasis.

Risk factor	Mechanism/Clinical association	
Pregnancy-associated *Candida *colonization [30]	Linked to preterm birth, neonatal mortality, and invasive neonatal candidiasis	
Vertical transmission [30]	Up to 65% of colonized pregnant women transmit *Candida *to neonates	
Third trimester [30]	Highest prevalence of VVC is reported during this period	
Elevated oestrogen levels [30], [31]	Reduce vaginal immunoglobulins and impair epithelial inhibition of *Candida *growth	
Elevated progesterone levels [30]	Suppresses neutrophil anti-*Candida *activity	
Emotional stress during pregnancy [31]	Suppresses immune responses, promoting *Candida *pathogenicity	
Reduced cell-mediated immunity [31]	Enhances susceptibility to *Candida *overgrowth	
Increased vaginal glycogen [31]	Provides a favourable substrate for *Candida *proliferation	
Low pH in the vagina [31]	Facilitates *Candida *colonization	
Diabetes mellitus [31]	Increases risk of *Candida *colonization in the vagina throughout pregnancy.	
HIV infection [31]	Immunosuppression predisposes to candidiasis	
The past history of candidiasis [31]	Associated with recurrent or persistent colonization	

Clinical manifestation

*Candida *spp. infection might cause superficial or deep-seated clinical symptoms [32]. Recurrent VVC typically manifests as persistent curdy white vaginal discharge, itching, an unpleasant odor, discomfort, and lower abdominal pain in 5-10% of pregnant women. Miscarriage, low birth weight, early membrane damage, and premature babies are all possible consequences of VVC [33]. Pruritus, hyperaemia, vaginal discomfort and leucorrhoea, burning, soreness, dyspareunia, and vaginal or vulvar erythema are the most typical clinical signs of VVC, which might interfere with marriage and sexual interactions [34]. Thick, curdy white discharge is a symptom of VVC [7]. Pelvic inflammatory disease (PID), infertility, pregnancy problems, recurrent UTIs, cervicitis, endometritis, and rising incidence of STDs are among the consequences that can result from these illnesses [7]. Some patients may experience vulvar symptoms rather than vaginal VVC signs. Other typical symptoms include thick vaginal discharge, oedema, fissures, and vulval and vaginal erythema [35].

*Candida *species associated with vaginitis

Most human infections caused by fungal pathogens are caused by species of *Candida *[36]. These species include the drug-resistant *C. glabrata,* a novel worldwide public health issue, *C. auris*, the most frequent reason for opportunistic infections, *C. albicans*, and other developing species, including *C. tropicalis, C. parapsilosis, and C. krusei* [36]. *C. albicans* is the most commonly encountered strain of *Candida *that causes vaginal candidiasis, followed by *C. glabrata, C. tropicalis, *and *C. parapsilosi*s [37].

C. albicans

As a benign commensal, *C. albicans* commonly resides in the oral, vaginal, and gastrointestinal mucosa of healthy people [25]. The polymorphic species *C. albicans*, which is able to develop as hyphae, pseudo hyphae, or yeast cells, is the most common reason for candidiasis [38]. Nonetheless, it’s generally recognized that hyphal cells are more adapted for tissue invasion and yeast cells for dissemination [25]. The pathogenicity of *C. albicans* requires both hyphal and yeast morphologies [25]. These include proteins involved in oligopeptide and iron transfer, secreted aspartyl proteases (SAPs) and phospholipases, which enable the breakdown of host barriers and the invasion of surrounding tissue, and ALS (agglutinin-like sequence) adhesins, which are required for host adhesion [39].

C. glabrata

This species' lack of pseudo hyphal development led to its first classification in the genus *Torulopsis*. It was suggested in 1978 that *Torulopsis glabrata* could be categorized within the genus *Candida *due of its connection to human infection when it was discovered that the capacity to produce pseudo hyphae was not a reliable differentiator for members of the genus [40]. Due to its innately lower susceptibility to azoles and its capacity to quickly develop azole resistance, this species continues to be among the most challenging to treat. Compared to other species of *Candida*, it is more closely linked to *Saccharomyces cerevisiae* [41]. As an alternative to *C. albicans,* it has been revealed that *C. glabrata* allows itself to be taken up by macrophages, where it endures and multiplies for extended periods of time before the fungal load causes cell lysis [39]. It can detoxify oxidative radical species and interfere with normal phagosomal maturation, which inhibits the production of phagolysosomes and the acidity of phagosomes [39].

C. tropicalis

In terms of genetic similarities, *C. tropicalis* is most related to *C. albicans*, whereas *C. glabrata* is least close [40]. In 1910, *C. tropicalis* was first identified as *Oidium tropicale* after being isolated from a patient suffering from fungal bronchitis [42]. It's a frequent reason for infections contracted in hospitals [43].

C. parapsilosis

*C. parapsilosis* continued to be a significant nosocomial and opportunistic infection that is typically treatable with antifungal medications [41]. Although this species cannot form real hyphae, it can develop pseudo hyphae, which are enormous and curved and are typically referred to as 'giant cells' [40]. This is linked to both localized and deep-seated infections, especially in newborns, and has a lower pathogenic potential than other species of *Candida *[43]. Vaginitis is a superficial infection that is clinically identical to a more prevalent* C. albicans* infection [43].

C. dubliniensis

A closely related organism that shares many phenotypic traits with *C. albicans *is *C. dubliniensis* [41]. This was given the name 'dubliniensis', since it was initially isolated from AIDS patients in Dublin, Ireland, in 1995, and is currently isolated all over the world. Its significance stems from the fast emergence of resistance to fluconazole, a major antifungal medication used to treat mycoses [43]. Furthermore, as previously mentioned, *C. dubliniensis* is the only species of *Candida* that can form hyphae aside from *C. albicans* [24].

C. krusei

It was previously discovered that this species is naturally resistant to most of antifungal medications, especially derivatives of azoles [43]. Its natural resistance to fluconazole and decreased sensitivity to AMB are well known [41]. It produces pinkish to purplish-coloured colonies on CHROMagar *Candida *medium, which might be mistaken for *C. glabrata *because of their comparable pink colony production [41].

C. auris

This organism often shows multidrug resistance and might be difficult to identify using conventional microbiologic techniques. Since the first occurrence eight years ago, *C. auris* has emerged as a hazard to global health, linked to several invasive infections and epidemics in medical facilities. India, South Africa, Kuwait, the United Kingdom, Venezuela, Brazil, the United States, Colombia, Pakistan, Spain, Germany, Israel, Norway, and Oman are among the countries where instances have been found thus far [44]. *C. haemulonii *and *C. ruelliae* are phylogenetically related to *C. auris* [44]. From vaginal secretions, *C. auris *has been identified and linked to vulvovaginitis in a clinical setting [44].

Virulence factors

The capacity of *C. albicans* to transition between yeast and filamentous growth forms is one of its most significant and well-researched virulence characteristics; this characteristic is also shared by *C. dubliniensis* [24]. Adherence to the vaginal epithelium follows colonization, and then virulence factors cause invasion, infection, and inflammation. Certain *Candida* virulence factors such as lipases, proteases, and candidalysin allow yeasts to cause infection [12]. Many virulence mechanisms, including adhesion to host tissues and medical devices, biofilm development, and generation of extracellular hydrolytic enzymes, assist the transformation pertaining to *Candida *species from commensal to potent pathogen [22].

The complicated structure of the cellular membrane includes mannans, phosphomannans, and chitin in addition to glucan; nevertheless, various species of *Candida *exhibit varying levels of mannan complexity and glucan exposure. Mannans are obtained in the outer layer, whereas beta-glucans and chitin are located in the innermost layer. Because of this structure, mannan is crucial in lowering the immunogenic exposure of glucan; nevertheless, *C. albicans* has additionally been revealed to exhibit coordinated chitin and glucan exposure [39]. Adhesins (like Hwp1 and the Als family), extracellular enzymes (like the secreted aspartyl proteinase (Sap) family and phospholipases), and - above all - the capacity to switch between unicellular yeast and filamentous hyphal forms of growth are the most frequently mentioned *C. albicans* virulence factors [24].

*C. albicans* must induce distinct stress resistance responses in response to the numerous stressors placed on it by host immune cells and the varied microenvironmental cues. For instance, *C. albicans* has six superoxide dismutase’s (SODs), which are all engaged in reactive oxygen species (ROS) detoxification by converting O₂ into molecular oxygen and hydrogen peroxide in the interest of evading oxidative death by immune cells. SOD-deficient strains of *C. albicans *are hence less virulent. Oxidative stress is lessened by more fungal antioxidant proteins and DNA damage repair genes [36]. Furthermore, strains of* C. parapsilosis*, as well as *C. tropicalis *that were modified to display hyper-filamentation phenotypes by constitutively expressing the transcriptional regulator UME6, demonstrated a notable decline in the organ fungal burden during in vivo infection [36].

The ECE1 gene encodes the pore-forming α-helical peptide toxin known as candidalysin, which is also expressed by *C. albicans. *which produces the protein Ece1p [36]. Kex2p and Kex1p metabolize Ece1p to produce mature candidalysin, which is subsequently secreted [36]. Candidalysin-induced holes in the host cell membrane likely allow cytoplasmic contents to seep into the invasion pocket [25]. The resulting calcium influx and oxidative stress in host cells cause rapid necrotic cell death instead of apoptotic cell death, giving the fungus more nutrients [36]. This could involve having access to vital micronutrients like zinc and iron [25]. From pH 2 to pH 10, *C. albicans* may flourish in a remarkably broad range of ambient pHs [25]. Given that *C. albicans *can colonize host niches with varying pHs, such as the vagina (acidic), GI tract (acidic to mildly alkaline), and blood (neutral), pH responses are very important [25]. The solubility of trace metals is similarly influenced by ambient pH, and, as a result, *C. albicans* regulates its micronutrient assimilation mechanisms in response to pH [25].

Pathogenesis

*C. albicans* must first be detected at the mucosal contact before an immune response may begin. Pattern recognition receptors (PRR), which are found on the cellular surface of epithelial and innate immune cells, are ultimately in the role of mediating this [45]. When it comes to VVC, the roles of the Toll-like receptor (TLR) and type C lectin receptor (CLR) families constitute the best understood [45]. Hyphae are vital for inflicting harm on tissue and penetrating mucosal barriers. Degradative enzymes released at the hyphal tip can weaken host membranes, allowing the elongating filament to exert enough pressure to penetrate the host cell [45]. Since its gene product is critical for pathogenicity, the ECE1 (extent of cell elongation) locus of *C. albicans* has drawn tremendous interest [45]. Candidalysin exacerbates innate immune signalling by causing excessive damage to mucosal surfaces [45]. Protein degradation leading to tissue invasion is triggered by the proteinase enzyme [39].

When *C. albicans* is functioning, it programs to survive the acidic, nutrient-poor environment so as to evade clearance within phagosomes. Fungal cells activate metabolic starvation pathways such as gluconeogenesis, fatty acid breakdown, and downregulation of translation in a bid to adapt to this nutrient-poor habitat. Ammonia is formed by yeast cells to cause filamentation, which facilitates the alteration from yeast to hyphae and neutralizes the acidic phagosomal pH [36].

Microbiome-immune dynamics in VVC

The mucosal epithelial barrier, endocrine modulation, and appropriate vaginal flora make up the vaginal microenvironment [28]. Healthy women's vaginas are colonized by a variety of microorganisms, with *Lactobacillus *accounting for 95% of these [28]. Oestrogen and progesterone cause periodic changes in vaginal epithelial cells. This process produces glycogen, which gives *Lactobacillus *energy to thrive [28]. Doderlein published the first comprehensive study on vaginal flora in 1894. The local environment and hormones have a significant impact on the typical flora of the vagina [46].

Through a number of different ways, Lactobacilli are known to operate antagonistically with *C. albicans*. Through competition for nutrients and adhesion sites, growth and hyphae formation suppression, and the excretion of fungicidal and fungistatic chemicals, these bacteria suppress *C. albicans* [47]. Additionally, by preventing the progression of hyphae, Lactobacilli reduced the pathogenicity of *C. albicans.* A low pH caused by Lactobacilli's synthesis of diverse short-chain fatty acids (SCFAs) inhibited filamentation [47].

*Lactobacillus *species often predominate in the vaginal bacterial microbiome of healthy women of reproductive age. These microbes synthesize acid known as lactic acid, which helps maintain a healthy vaginal pH that is typically less than 4.5. The five main categories of community state types (CSTs) of the microbial flora of the vagina consist CST-I (dominated by *Lactobacillus crispatus*), CST-II (dominated by *Lactobacillus gasseri*), CST-III (dominated by* Lactobacillus iners*), and CST-V (dominated by *Lactobacillus jensenii*). Compared to the other categories, the CST-IV state is incredibly varied, including microbial agents linked to BV and anaerobes. Further subgroups of CST-IV have been identified: CST-IVA (containing some Lactobacilli), CST-IVB (high prevalence of *Atopobium *spp.), CST-IVC (dominated by *Gardnerella *subgroup A), and CST-IVD (related with *Gardnerella* subgroup C) [25].

The term 'vaginal dysbiosis' (VD) refers to a disruption in the microbial balance of the vagina, which is typified by a decline in beneficial *Lactobacillus *species. This weakens the defences of the vaginal epithelium and permits the proliferation of pathogens [48]. As a result, diseases including trichomoniasis, vaginal candidiasis, and desquamative inflammatory vaginitis are also regarded as forms of VD [48]. The placenta produces oestradiol and estriol during a healthy pregnancy, which raises *Lactobacillus *levels and lowers vaginal pH. Throughout pregnancy, the mother and foetus are shielded from infections by a steady Lactobacilli population that is sustained by an acidic pH. Additionally, pregnant women have a different vaginal bacterial composition than non-pregnant women with higher concentrations of *Clostridium *and *Bacteroides* [48].

By shedding the superficial epithelial layer into the vaginal lumen and coating epithelial cells with mucin, vaginal epithelial cells restrict *C. albicans* from binding to and invading the mucosa. Both *C. albicans* and non-albicans species (such as *C. glabrata, C. parapsilosis, *and *C. tropicalis*) share a protective type I interferon response that is triggered by early mitochondrial signalling upon fungal sensing. This is followed by a damage response that is unique to *C. albicans* and is controlled by candidalysin secretion [36]. The host actively monitors and defends its barrier surfaces through two separate, complementary, and cooperating branches of the immune system: innate and adaptive immunity. This prevents microbial overgrowth on epithelial barriers and microbial infiltration of tissues [25].

*Candida *speciation

Targeted therapy is made possible by accurate speciation, which guarantees the selection of the best antifungal medication and reduces the chance of treatment failure. Because of the frequent involvement of NAC species, which are generally more opposing to conventional treatments, speciation is even more crucial in recurrent or chronic VVC. When resistance to conventional therapies is found, early intervention is made possible by identifying the particular species, which also aids in developing therapeutic strategies [49].

In clinical laboratories, a germ tube test usually serves as the first step in yeast identification operations. It is a quick way to distinguish *C. albicans *and *C. dubliniensis* from other species of *Candida*. Despite being a quick test, false positive and false negative findings are possible. Supplementary testing takes place, including carbohydrate fermentation, carbohydrate absorption, and culture on cornmeal agar. Growth patterns on cornmeal agar can be found in 24-72 hours, while tests for sugar assimilation can take from 72 hours to two weeks. Determining the diagnosis and selecting the appropriate antifungal medication are labour-intensive processes that take longer [50].

CHROMagar *Candida *differential agar

A number of chromogenic substrates holding culture medium have been created to enable quick identification. These unique media produce microbial colonies with different colours as a result of chromogenic substrates reacting with microorganism-secreted enzymes [50]. Chromogenic media were developed in response to the difficulty of rapidly recognizing colonies on conventional Sabouraud's dextrose agar (SDA). In addition to aiding in the quick detection of yeast infections from clinical samples, this development yields results 24-48 hours ahead of time when compared to conventional identification techniques. A useful tool in the lab for the presumed speciation of *Candida *in yeast infections is CHROMagar *Candida *(HiMedia, Mumbai, India) agar [49]. *Candida *species such as *C. albicans, C. krusei, C. tropicalis, C. glabrata, C. parapsilosis, *and *C. dubliniensis*, as well as other yeast-like colonies can be simultaneously isolated and presumed to be identified using this method [43].

Based on their distinct metabolic characteristics and colony appearance, these media allow for the separation and probable identification of *Candida *species. CHROMagar has substrates that undergo a reaction with particular enzymes made by several species of *Candida*, giving colonies unique colours [49]. This medium could potentially replace cornmeal agar and conventional biochemical assays utilized for direct identification of *C. albicans*, such as germ tube tests, sugar fermentation tests, and sugar assimilation tests, due to the usual colour displayed by *Candida *species [50].

Compared to traditional methods that are expensive, time-consuming, and technically challenging, chromogenic agar is quick, easy, and affordable [27]. It offers a precise way to identify *Candida *species, which helps choose the right course of treatment and enhances patient outcomes in cases of antifungal resistance [49].

After 48-72 hours of incubation at 30°C, colonies on CHROMagar *Candida *exhibit characteristic colour variations*, *as shown in* *Table 3 [43].

**Table 3:** Candida colony colour on CHROMagar. Source: Created by the author based on reviewed literature.

*Candida *species	Colour of colonies
C. albicans	Light green
C. dubliniensis	Dark green
C. glabrata	Pink to purple
C. krusei	Pink
C. parapsilosis	Cream to pale pink
C. tropicalis	Blue with pink halo [43]

Antifungal resistance and therapeutic implications in *Candida*

The reduction of growth in vitro relative to drug-untreated cells is a measure of antifungal activity. Two main methods from AFST subcommittees, such as the European Committee on Antimicrobial Susceptibility Testing (EUCAST) and the Clinical Laboratory Standards Institute (CLSI) are used to determine antifungal susceptibility [51]. The goals of VVC treatment are to eradicate the infection, reduce symptoms, and stop recurrence. Topical antifungals, such as over-the-counter and prescription creams, ointments, suppositories, and tablets containing compounds including clotrimazole, miconazole, and tioconazole are the main therapeutic choice. For severe or recurrent infections, oral antifungals like fluconazole are used. A single dose is frequently effective, while longer courses may be necessary in chronic situations [49]. Due to toxicity, growing medication resistance, and drug-drug interactions, the current standard antifungal treatments are limited [52].

Consequently, they are categorized as either triazoles (itraconazole, fluconazole, and terconazole) or imidazoles (ketoconazole, miconazole, clotrimazole, econazole, and butoconazole) [53]. By acting on the cytochrome P450-dependent enzyme lanosterol 14α-demethylase, the azoles prevent ergosterol formation in the fungal cell membrane [53]. AMB, the erstwhile 'gold standard' antifungal, always induces toxicity in patients, undermining its efficacy as a fungicidal agent. It does this by binding to the fungal membrane component ergosterol and altering membrane permeability [52]. Fluconazole, a participant in the azole class of antifungals, is the drug that is consistently prescribed for *C. albicans* infections [54]. Treatment for *Candida *infections consists of a wide variety of antifungal medications [32]. These consist of nucleoside analogues, azoles, polyenes, and echinocandins [32].

*C. albicans* absorbs 5-fluorocytosine (5-FC), a pyrimidine analogue, and transforms it into 5-fluorouracil, which prevents the synthesis of fungal DNA and RNA [36]. The azoles target Erg11p/Cyp51 and block ergosterol biosynthesis, which causes toxic sterols such 14α-methylergosta-8,24(28) dienoyl to accumulate on fungal cell membranes and increases endogenous ROS levels; they both aid in fungal growth arrest. Imidazoles are applied topically to treat mucosal candidiasis [36]. The parenterally administered echinocandins, which are fungicidal and comprise the well-tolerated caspofungin, micafungin, and anidulafungin, inhibit β-(1,3)-glucan synthase, an enzyme involved in the generation of β-(1,3)-glucan. In the absence of β-(1,3)-glucan, fungal cell wall rigidity is lost, leading to cell lysis [36,52].

When fungi acquire the capacity to proliferate in the presence of antifungal medications that would typically eliminate them or hinder their growth, it is known as antifungal resistance [32]. But most triazoles are fungistatic rather than fungicidal, which makes it possible for resistant mutants to arise [52]. Drug exposure or medication can result in an emergence of antifungal resistance in vitro [51]. NAC species frequently exhibited opposition to the azole category of antifungal medicine [22]. Although the clinical signs of infections caused by several NAC species are typically indistinguishable, some NAC species are either naturally resistant to routinely used antifungal medications, capable of developing resistance to them, or both [22].

Up to 7.5% of *Candida *species from the vaginal area are resistant to at least one of the widely used azoles based on the findings [53]. AMB resistance in *C. albicans* is extremely rare and is linked to mutations in the ergosterol production pathway's ERG3 or ERG6 genes, which lower ergosterol levels and prevent AMB from attaching to the fungus's cell wall [36]. The azole systems for resistance in *C. tropicalis* are comparatively poorly understood as contrasted with other species of *Candida*. Furthermore, a subset of fluconazole-resistant isolates that are additionally tolerant to itraconazole and voriconazole had even greater levels of ERG11 expression [54].

The elevated expression of ERG11 resulting from activating mutations in the gene encoding the zinc-cluster transcriptional regulator Upc2p is another contributory factor of fluconazole resistance in *C. albicans* [54]. The upregulation of the drug efflux pumps Mdr1p and Cdr1p/Cdr2p is one of two additional mechanisms of fluconazole resistance in *C. albicans*. The zinc-cluster transcription factor TAC1 (transcriptional activator of CDR genes) is identified by the ATP-binding cassette (ABC) transporter-associated genes CDR1 and CDR2 [54]. Mdr1p is a major facilitator superfamily (MFS) efflux pump that is persistently increased in certain fluconazole-resistant *C. albicans* isolates and often expressed at undetectable levels in wildtype *C. albicans* strains. It is also activated in the presence of benomyl, diamide, and hydrogen peroxide [54].

It is alarming that echinocandin-resistant clinical strains of *C. albicans* have become more prevalent in recent years. Mutations in core regions of the β-(1,3)-glucan synthase gene FKS are the most common pathway of resistance, and they are usually linked to previous exposure to echinocandins. Furthermore, but more common in *C. glabrata* [36], almost all current antifungal medications are significant, and it has been demonstrated that fungus-related illnesses have acquired antifungal resistance [51].

Clinical guidelines and management ​​​​​​of recurrent vaginitis

Probiotics are a type of helpful microorganism that live in the human reproductive and digestive systems. Probiotics taken orally have been proven in numerous trials to be proficient at managing a wide range of digestive system disorders. The vaginal microecological homeostasis can be altered or maintained by *Lactobacillus*, the most prevalent bacterial species in the vaginal milieu [28].

Probiotics are useful in the short term for treating common vaginal infections in non-pregnant adult women when used in conjunction with traditional pharmaceutical therapies. Yet there is little high-quality data encouraging the supplementation of probiotics alone to treat or prevent recurrent vaginal infections. Additional detailed clinical investigation is required to figure out the most efficient probiotic strains, the optimal treatment protocols (with or without antibiotics), and the specific female subgroups (e.g., premenopausal vs. postmenopausal) that are likely to gain the greatest benefit from probiotic therapy [29].

Lactobacilli are frequently employed as probiotics due to their diverse antagonistic effects on numerous infections. Freeze-dried Lactobacilli put on applications, capsules and tampons allow for immediate access to the vaginal tract. Lactobacilli-containing capsules or foods like yogurt allow for oral delivery through the gastrointestinal tract (GI tract). Numerous studies revealed that probiotics containing Lactobacilli increased the number of Lactobacilli in healthy women. Probiotics improved the effectiveness of azole treatment in VVC patients by decreasing fungal colonization. Relapse was avoided and a long-term cure resulted. Additionally, probiotic therapy enhanced the subjective alleviation of itchy and painful symptoms [47].

Social and economic impact of *Candida *vaginitis

*Candida *vaginitis is a globally significant illness that has profound consequences on women's general psychological and physical wellness as well as the medical and labour economies [45]. In contrast, VVC results in an adverse impact on the individual's professional and community life. Since consuming foods high in sugar, carbs, yeasts, or dairy-based products has been linked to enhanced growth of fungus, some researchers think that nutrition is contributing to the development of VVC [12]. In spite of having a negative impact on a patient's bodily and mental well-being, this illness can cause fertility issues, substantial monetary expenses, and problems in marriage [55].

Research gap and future directions

In order to mitigate the burden of this unsatisfied clinical demand, new treatment modalities, enhanced diagnostics, and extensive pathogenomics investigations will be necessary, since the incidence of VVC is expected to increase by tens of millions worldwide over the next 10 years [45]. Because vaginitis complaints are non-specific, a diagnosis made without test confirmation may result in the wrong treatment being prescribed [23].

Public health is especially concerned about the gradual epidemiologic change from species susceptible to azole antifungals to resistant ones [20]. The development of more effective methods for detection and management of afflicted individuals should be made possible by a knowledge of the virulence characteristics of *C. albicans*, the tissue-specific mechanisms of anti-*Candida *host defences, and its mechanisms of resistance to the arsenal of currently available antifungal medications [36].

In addition, the discovery of clinical isolates of *C. glabrata* that are resistant to multiple drugs (azole and echinocandin) raises serious concerns, since there aren't many and highly hazardous therapeutic choices available for individuals infected with these strains. Therefore, the urgent need to find novel antifungal medication classes is highlighted by the small number of current antifungal drug classes, the rising incidence of bloodstream fungal infections, and the emergence of antifungal drug resistance [52].

Limitations

This review has several limitations that should be considered while interpreting the findings. First, only English-language articles were included, which may introduce language bias and limit the inclusion of relevant studies published in other languages. Second, although a structured approach based on PRISMA principles was used, this is not a fully systematic review and lacks a formal risk-of-bias assessment.

Additionally, the included studies show considerable heterogeneity in terms of study design, population characteristics, diagnostic methods, and AFST techniques, making direct comparisons difficult. Most of the available data are derived from single-centre or regional studies, which may not be representative of broader populations and thus limit generalizability.

Furthermore, a significant proportion of the evidence on antifungal resistance is based on in-vitro susceptibility data, with limited correlation to clinical outcomes such as treatment failure or recurrence. The absence of large-scale, multicentric, longitudinal studies further restricts the ability to draw definitive conclusions regarding resistance trends and their clinical impact.

## Conclusions

VVC is a widespread gynaecological infection with significant clinical as well as societal wellness impacts. Although *C. albicans *remains the predominant causative agent, infections due to NACs are increasing and are often linked to decreased susceptibility to commonly used antifungal agents. The progression of VVC is multifactorial, involving host factors, hormonal influences, immune status, and disruption of the normal vaginal microbiota. Accurate screening of species and tolerance to fungal medication testing are therefore essential, particularly in recurrent and complicated cases, to guide effective therapy. Given the rising antifungal resistance and the emergence of difficult-to-treat *Candida *species, continued surveillance and improved diagnostic strategies are essential for maximizing management and reducing disease burden.

## References

[1] Budhani D, Mehta S, Rudra S, Kumar A (2016). Isolation, identification and anti-fungal susceptibility of candida species from clinically suspected cases of vulvovaginitis in a tertiary care hospital in rural area—a cross-sectional study. Indian J Microbiol Res.

[2] ElFeky DS, Gohar NM, El-Seidi EA, Ezzat MM, AboElew SH (2016). Species identification and antifungal susceptibility pattern of candida isolates in cases of vulvovaginal candidiasis. Alexandria J Med.

[3] Paul P, Das P, Pandey N (2025). Vulvovaginal candidiasis: characterization of etiologic agent with their antifungal susceptibility pattern. Int J Health Sci Res.

[4] Kombade SP, Abhishek KS, Mittal P, Sharma C, Singh P, Nag VL (2021). Antifungal profile of vulvovaginal candidiasis in sexually active females from a tertiary care hospital of Western Rajasthan. J Family Med Prim Care.

[5] Bashir G, Altaf I, Khurshid R, Ahmed T, Ali A, Zaffar S (2023). Identification and pattern of antifungal susceptibility of Candida species isolated from cases of vaginitis in a tertiary care hospital in India. Iran J Microbiol.

[6] Hoare BS, Mikes BA, Khan YS (2026). Anatomy, abdomen and pelvis: female internal genitals. https://www.ncbi.nlm.nih.gov/sites/books/NBK554601/.

[7] Jayalaxmi M, Humera A (2019). A clinicoetiological study of vulvovaginitis in a tertiary care hospital. Int J Reprod Contracept Obstet Gynecol.

[8] Venugopal S, Gopalan K, Devi A, Kavitha A (2017). Epidemiology and clinico-investigative study of organisms causing vaginal discharge. Indian J Sex Transm Dis AIDS.

[9] Rosner J, Samardzic T, Sarao MS (2026). Physiology, female reproduction. https://www.ncbi.nlm.nih.gov/books/NBK537132/.

[10] Pan Z, Wu Y, Li Y, Hu X, Zhao Y, Liu Y (2023). Retrospective study of pathogens involved in vaginitis among Chinese women. BMC Womens Health.

[11] Bhargava D, Kar S, Saha A, Saha M (2016). Prevalence of vaginitis in females attending national medical college and teaching hospital, Birgunj, Nepal. Indian J Med Res Pharm Sci.

[12] Farr A, Effendy I, Tirri BF (2021). Guideline: vulvovaginal candidosis (AWMF 015/072, level S2k). Mycoses.

[13] Samal R, Vaithy A, Kotasthane DS, Ghose S (2015). Prevalence and clinico-mycological profile of vulvovaginal candidiasis in a tertiary care hospital. Int J Reprod Contracept Obstet Gynecol.

[14] Makanjuola O, Bongomin F, Fayemiwo SA (2018). An update on the roles of non-albicans Candida species in vulvovaginitis. J Fungi (Basel).

[15] Thulkar J, Kriplani A, Agarwal N, Vishnubhatla S (2010). Aetiology & risk factors of recurrent vaginitis & its association with various contraceptive methods. Indian J Med Res.

[16] Bitew A, Abebaw Y (2018). Vulvovaginal candidiasis: species distribution of Candida and their antifungal susceptibility pattern. BMC Womens Health.

[17] Mariyah S, Iyer RN, Jangam RR, Kesireddy S (2022). Vulvovaginal candidiasis: clinical profile, species distribution and antifungal susceptibility pattern. J Acad Clin Microbiol.

[18] Bradford LL, Ravel J (2017). The vaginal mycobiome: a contemporary perspective on fungi in women's health and diseases. Virulence.

[19] Ghaddar N, Anastasiadis E, Halimeh R (2020). Prevalence and antifungal susceptibility of Candida albicans causing vaginal discharge among pregnant women in Lebanon. BMC Infect Dis.

[20] Uppuluri P, Khan A, Edwards JE (2017). Current trends in candidiasis. Candida albicans: Cellular and Molecular Biology.

[21] Rahate K, Arshi A, Barai RS, Chakraborty S, Idicula-Thomas S (2024). EpiCandIn: an open online resource for epidemiology of Candida infections in India. Indian J Med Res.

[22] Deorukhkar SC, Saini S, Mathew S (2014). Non-albicans Candida infection: an emerging threat. Interdiscip Perspect Infect Dis.

[23] Narayankhedkar A, Hodiwala A, Mane A (2015). Clinicoetiological characterization of infectious vaginitis amongst women of reproductive age group from Navi Mumbai, India. J Sex Transm Dis.

[24] Moran GP, Coleman DC, Sullivan DJ (2012). Candida albicans versus Candida dubliniensis: why is C. albicans more pathogenic?. Int J Microbiol.

[25] d'Enfert C, Kaune AK, Alaban LR (2021). The impact of the Fungus-Host-Microbiota interplay upon Candida albicans infections: current knowledge and new perspectives. FEMS Microbiol Rev.

[26] Msomi N, Sukali G, Shangase C, Mabaso N, Abbai N (2024). An overview of vaginitis and vulvovaginal health. J Med Lab Sci Technol S Afr.

[27] Waikhom SD, Afeke I, Kwawu GS (2020). Prevalence of vulvovaginal candidiasis among pregnant women in the Ho municipality, Ghana: species identification and antifungal susceptibility of Candida isolates. BMC Pregnancy Childbirth.

[28] Mei Z, Li D (2022). The role of probiotics in vaginal health. Front Cell Infect Microbiol.

[29] Jeng HS, Yan TR, Chen JY (2020). Treating vaginitis with probiotics in non-pregnant females: a systematic review and meta-analysis. Exp Ther Med.

[30] Tsega A, Mekonnen F (2019). Prevalence, risk factors and antifungal susceptibility pattern of Candida species among pregnant women at Debre Markos Referral Hospital, Northwest Ethiopia. BMC Pregnancy Childbirth.

[31] Disha T, Haque F (2022). Prevalence and risk factors of vulvovaginal candidosis during pregnancy: a review. Infect Dis Obstet Gynecol.

[32] Cortegiani A, Misseri G, Fasciana T, Giammanco A, Giarratano A, Chowdhary A (2018). Epidemiology, clinical characteristics, resistance, and treatment of infections by Candida auris. J Intensive Care.

[33] Panda PS, Muralidhar S, Lachyan A (2024). Fluconazole resistance in candida isolates from vaginal discharge in women attending an apex regional sexually transmitted infections center. Saudi J Health Sci.

[34] Kumari V, Banerjee T, Kumar P, Pandey S, Tilak R (2013). Emergence of non-albicans Candida among candidal vulvovaginitis cases and study of their potential virulence factors, from a tertiary care center, North India. Indian J Pathol Microbiol.

[35] Siddiqui R (2019). Clinical patterns and risk factors of vulvo-vaginal candidiasis among women of reproductive age attending a tertiary hospital in central India. Stamford J Microbiol.

[36] Lopes JP, Lionakis MS (2022). Pathogenesis and virulence of Candida albicans. Virulence.

[37] Nelson M, Wanjiru W, Margaret MW (2013). Prevalence of vaginal candidiasis and determination of the occurrence of candida species in pregnant women attending the antenatal clinic of Thika district hospital, Kenya. Open J Med Microbiol.

[38] Sullivan DJ, Moran GP, Pinjon E (2004). Comparison of the epidemiology, drug resistance mechanisms, and virulence of Candida dubliniensis and Candida albicans. FEMS Yeast Res.

[39] Galocha M, Pais P, Cavalheiro M, Pereira D, Viana R, Teixeira MC (2019). Divergent approaches to virulence in C. albicans and C. glabrata: two sides of the same coin. Int J Mol Sci.

[40] Silva S, Negri M, Henriques M, Oliveira R, Williams DW, Azeredo J (2012). Candida glabrata, Candida parapsilosis and Candida tropicalis: biology, epidemiology, pathogenicity and antifungal resistance. FEMS Microbiol Rev.

[41] Brandt ME, Lockhart SR (2012). Recent taxonomic developments with Candida and other opportunistic yeasts. Curr Fungal Infect Rep.

[42] Zuza-Alves DL, Silva-Rocha WP, Chaves GM (2017). An update on Candida tropicalis based on basic and clinical approaches. Front Microbiol.

[43] Chander J (2018). Textbook of Medical Mycology. Textbook of medical mycology.. Chander J (ed): Jaypee Brothers Medical.

[44] Sears D, Schwartz BS (2017). Candida auris: an emerging multidrug-resistant pathogen. Int J Infect Dis.

[45] Willems HM, Ahmed SS, Liu J, Xu Z, Peters BM (2020). Vulvovaginal candidiasis: a current understanding and burning questions. J Fungi (Basel).

[46] Prasad PV, Kaviarasan PK, Kannambal K, Nethra T (2016). Genital discharge in females-a review. Indian J Clin Exp Dermatol.

[47] Krüger W, Vielreicher S, Kapitan M, Jacobsen ID, Niemiec MJ (2019). Fungal-bacterial interactions in health and disease. Pathogens.

[48] Dubé-Zinatelli E, Cappelletti L, Ismail N (2024). Vaginal microbiome: environmental, biological, and racial influences on gynecological health across the lifespan. Am J Reprod Immunol.

[49] Balaji L, Subramaniam J (2024). Bridging diagnostic gaps: utilising HiCrome agar and tetrazolium reduction medium for the rapid and presumptive identification and speciation of Candida species in vulvovaginal candidiasis in low-resource environments. Cureus.

[50] Baradkar VP, Mathur M, Kumar S (2010). Hichrom Candida agar for identification of Candida species. Indian J Pathol Microbiol.

[51] Sanglard D (2017). Mechanisms of drug resistance in Candida albicans. Candida albicans: Cellular and Molecular Biology.

[52] Chauhan N (2017). Signaling mechanisms in pathogenesis and virulence of Candida albicans. Candida albicans: Cellular and Molecular Biology.

[53] Alikhani T, Daie Ghazvini R, Mirzaii M (2022). Drug resistance and biofilm formation in Candida species of vaginal origin. Iran J Public Health.

[54] Whaley SG, Berkow EL, Rybak JM, Nishimoto AT, Barker KS, Rogers PD (2016). Azole antifungal resistance in Candida albicans and emerging non-albicans Candida species. Front Microbiol.

[55] Hedayati MT, Taheri Z, Galinimoghadam T, Aghili SR, Yazdani Cherati J, Mosayebi E (2015). Isolation of different species of Candida in patients with vulvovaginal candidiasis from Sari, Iran. Jundishapur J Microbiol.

